# Shapelet selection based on a genetic algorithm for remaining useful life prediction with supervised learning

**DOI:** 10.1016/j.heliyon.2022.e12111

**Published:** 2022-12-07

**Authors:** Gilseung Ahn, Min-Ki Jin, Seok-Beom Hwang, Sun Hur

**Affiliations:** a Big Data Group, Hyundai Motors Company, Seoul, 06796, Republic of Korea; b Dept. of Industrial & Management Engineering, Hanyang University, Ansan, 15588, Republic of Korea

**Keywords:** RUL shapelet selection, Remaining useful life prediction, Genetic algorithm, Feature selection

## Abstract

RUL (remaining useful life) shapelets were recently developed to overcome the shortcomings of similarity-based RUL prediction methods, such as high sensitivity to parameters. RUL shapelets are informative subsequences whose distances to a run-to-failure time series sample are very useful for predicting the RUL of the sample. However, the prediction performance and interpretability highly depend on the set of RUL shapelets, and it is very difficult to compose an optimized set. In this paper, we mathematically formalize the RUL shapelet composition problem with multiple objective functions. In addition, we analyze the characteristics of good RUL shapelet sets and develop a solution methodology based on a genetic algorithm. From the various experiments, we validate that the proposed method outperforms previous ones and suggest how to use the proposed method. The solution methodology developed in this paper can be applied to solve various RUL prediction problems. In addition, the findings on the RUL shapelets can help researchers develop their RUL shapelet-based solution.

## 1 Introduction

The remaining useful life (RUL) of an engineering system is defined as the length of time from the current time to the time of failure. It is essential for prognostics and health management (PHM) to predict the RUL accurately because the predicted RUL contributes to make important decisions such as maintenance schedules. In other words, PHM objectives such as avoiding accidents, anticipating failures and aiming reliable operation and maintenance can be obtained through accurate RUL prediction (Zio, 2022; Zonta et al., 2020).

RUL prediction approaches can be categorized into physics-based and data-driven approaches. The former relies on developing degradation process models to estimate the RUL by using domain knowledge, such as system failure mechanisms, while the latter relies on developing data-driven models by discovering degradation patterns from previously observed data of the system and estimating RUL based on these patterns by using statistical machine learning (ML) or deep learning (DL) models (Liao and Kottig, 2014). In this paper, we focus on the data-driven approach with ML or DL models, which has attracted much attention from both academia and industry thanks to the recent development of data collection and processing techniques.

Methods to develop RUL prediction models using ML or DL models can be categorized into various categories, including statistical feature-based, image-based, time series-based, and similarity-based models. Statistical feature-based methods extract statistics from a run-to-failure time series and use them as a feature vector (Guo et al., 2017; Aremu et al., 2020). Image-based methods convert run-to-failure data into 2D images using time-frequency representation techniques and train patterns from the images using convolution neural network models (Yoo and Baek, 2018). Time series-based methods construct health index and train time series models such as long short-term memory (LSTM) using the health index to predict RUL (Xiang et al., 2021; Sharma et al., 2022). Similarity-based methods estimate RUL of newly entered time series samples based on the RUL values of nearest neighbors (Mosallam et al., 2016). To be more concrete, the methods split every training sample into a set of windows and labels RUL for each window. Then, the method finds the nearest neighbors of a window of new sample among training windows. Finally, the RUL is predicted as the weighted mean of RULs of the neighbors, where each weight is directly proportional to the similarity between the window and neighbor.

It is very important for RUL prediction models to consider not only prediction accuracy but also interpretability in order to use them for explainable diagnosis (Costa and Sánchez, 2022). RUL prediction results using similarity-based models are interpretable based on the neighbor windows. In addition, they are appropriate for dealing with run-to-failure data collected from various operating conditions (Li et al., 2017). Finally, it is easy to implement without any domain knowledge and analyzing degradation trends (Cai et al., 2020), because it employs similar historical data as references and relies on the historical data itself. In addition, it is difficult to obtain enough degradation data in real world applications (Ahn et al., 2021), but the similarity-based method has been proven effective to predict RUL with the limited data (Lyu et al., 2020).

Owing to these advantages, these methods have been frequently addressed in the literature such as Lyu et al. (2020), Liu et al. (2019), Bingjie et al. (2021) and Malinowski et al. (2015). For example, Lyu et al. (2020) proposed similarity-based RUL prediction method based on dynamic time warping (DTW). DTW is a distance measure to calculate distance between time series samples with different length. Since it requires huge computational time, they also introduce a coarse-to-fine strategy to find the neighbor windows in an efficient way. RUL of test window is estimated based on the neighbor windows and adjusted by degradation rate and time gap based adjustment strategies. Liu et al. (2019) developed an RUL prediction method that consists of three steps: (1) health index construction, (2) similarity matching, and (3) RUL prediction. In the first step, every multivariate training sample is transformed into a univariate health index using principal component analysis (PCA), and a new sample is also transformed similarly. In the second step, every health index is again transformed to a set of sliding windows of the same length. In the third step, the nearest neighbor of every window in the new sample is found among all windows in a training health index based on the mean of the Euclidean similarity and cosine similarity. Finally, the RUL of each window in the new sample is estimated as the weighted mean of RULs of its neighbor windows, where the weight is the sum of the normalized Euclidean and cosine similarities. Bingjie et al. (2021) employed K-means clustering algorithm for the similarity-based RUL prediction, considering that run-to-failure samples are collected under different operating conditions. That is, they use the algorithm to group similar training samples and find the closest cluster to each test sample. The training samples in the closet cluster are used to estimate RUL of the test sample using kernel density estimation.

Even though similarity-based methods have many advantages, they also have several critical disadvantages. For example, the prediction accuracy and interpretability are very sensitive to the hyperparameters, such as the window size, number of neighbors, and similarity measures. In addition, they usually use fixed window for low computational complexity, leading to miss potentially good windows. Using sliding window instead of the fixed window, however, leads to long computational time.

In this regard, Malinowski et al. (2015) proposed a method based on RUL shapelets by employing the concept of shapelets. Shapelets are the subsequences used for time series classification and distance between the shapelets and a time series sample are employ to determine its class (Ye and Keogh, 2009). It has been adopted in various applications owing to its several advantages including high accuracy, interpretability, few hyperparameters and so forth. For example, Liu et al. (2015) applied shapelets to recognize complex human activities suffering from portability, interpretability and extensibility. As another example, AlDhanhani et al. (2019) used shapelets to represent traffic incidents and congestion patterns for detecting traffic events. It is very difficult to find the optimal shapelets efficiently, and some studies addressed this problem. For example, Grabocka et al. (2014) applied stochastic gradient learning to find the near-to-optimal shapelets without evaluating a lots of shapelet candidates.

RUL shapelets are subsequences whose distance can be used as a feature vector to predict RUL. Since the RUL shapelets are more informative than window and the number of them is much smaller than that of windows, it is more effective and efficient to use RUL shapelets instead of similarity-based methods. Even though they proposed interesting concepts, RUL shapelets, they did not analyze the properties of the RUL shapelets and not consider interactions among the RUL shapelets. In addition, they predict RUL as a mean of RUL of time series samples after RUL shapelets appear. Therefore, it is necessary to consider the properties and interaction to use RUL shapelets effectively, and our research objective is to develop a RUL shapelet selection method considering them.

In order for the RUL shapelets to be used, the distance between time series samples and shapelets should be proportional to the RULs to obtain high accuracy. In addition, the number of shapelets should be small enough for the interpretability and to allow a short estimation time. Finally, there should be no redundancy among the RUL shapelets, positive interactions should exist among them. Here, redundant RUL shapelets are shapelets whose distances to time series samples are highly correlated with each other, and positive interaction between two RUL shapelets means that RUL can be estimated accurately only when considering both distances. It is obvious that the estimation accuracy, interpretability, and estimation time highly depend upon the set of selected RUL shapelets, but previous research did not consider this. Genetic algorithm (GA) is one of the most widely used metaheuristic algorithms to solve various time series data analysis problems. It has been successfully applied to solve various optimization problems including shapelet selection (Xue et al., 2020), which is similar to our problem.

The major contents and contributions of our research are as follows. First, we mathematically formulate the RUL shapelets selection problem as a feature selection problem with three objectives: (1) to minimize the error of RUL prediction, (2) to minimize the number of RUL shapelets, and (3) to minimize redundancy among the shapelets. To achieve this goal, we expand the concept of RUL shapelets to the feature vectors, by which the machine learning model can be trained in order to consider the interaction between RUL shapelets that appear in the different locations of the time series. Second, we discuss the properties of good RUL shapelets, including their redundancy and interactions. The discussion can be summarized as (1) selecting RUL shapelets considering correlation between distance to RUL shapelets and RULs only may lead to focusing on the later part of the time series, (2) good RUL shapelets occur at similar locations in every sample, and (3) even good RUL shapelets in the same interval causes redundancy which negatively impact on RUL prediction performance. Finally, we develop a GA to solve the formalized RUL shapelet selection problem. Especially, we focus on designing initialization method of the GA based on the discussion on the properties of good RUL shapelets. In addition, we also design proper fitness functions for our problem.

The rest of this paper is organized as follows. Section 2 presents the preliminaries of the study, including shapelet discovery, RUL shapelet, and GA. Section 3 introduces and formulates the RUL shapelets selection problem, and Section 4 analyzes the properties of the RUL shapelets and develops a GA-based RUL shapelet selection algorithm. Section 5 verifies the proposed algorithm through experiments. Finally, Section 6 concludes the research.

## 2 Preliminaries

### 2.1 Shapelet discovery

Let $x=\left(x_{1},x_{2},\cdots ,x_{T}\right)$ be a time series sample and y∈{1,2,⋯,C} be its label. We say $s=\left(s_{1},\;s_{2},\cdots ,s_{l}\right)$ is a subsequence of the time series dataset X∋x if there are one or more samples satisfying the following:

(1) $$s=x_{t:t+l},$$

where $x_{t:t+l}$ denotes $\left(x_{t},x_{t+1},\cdots ,x_{t+l}\right)$. The distance between arbitrary subsequence $s^{\prime }$ and ***x***, $d\left(s^{\prime },x\right)$, is defined as presented in equation (2).

(2) $$d\left(s^{\prime },x\right)=\min_{t=1,2,\cdots ,T_{i}-l}E\left(s^{\prime },x_{t:t+l}\right),$$

where E(a,b) is the Euclidean distance between two vectors, ***a*** and ***b***. We say $s^{\prime }$ matches $x_{t^{\prime }:t^{\prime }+l}$ when $x_{t^{\prime }:t^{\prime }+l}={\mathrm{argmin}}_{t=1,\;2,\;\cdots ,T_{i}-l}\;E\left(s^{\prime },x_{t:t+l}\right)$.

A shapelet is defined as the subsequence whose distance to each class maximizes the class relevance in time series classification problems (Ye and Keogh, 2009). In other words, the shapelet is the subsequence that minimizes the loss function of a classifier when its distance to each class is used as a feature as follows:

(3) $$s={\mathrm{argmin}}_{s^{\prime }\in S}\;L\left(f\left(d\left(s^{\prime },X\right)\right),y\right),$$

where L(⋅) is a loss function for a classifier *f*, *S* is a set of all possible subsequences, and ***y*** is the label vector.

Since the search space *S* is too big to find ***s*** in equation (3), many heuristic approaches have been proposed to find shapelets under several assumptions. For example, Grabocka et al. (2014) developed a learning method to estimate shapelets of a given length. The method initializes candidates of the shapelet randomly and updates them using stochastic gradient descent optimization to minimize the loss function. Note that shapelet ***s*** found by heuristic approaches would not satisfy equation (3). Even worse, ***s*** may not be the subsequence satisfying equation (1).

### 2.2 RUL shapelet

RUL shapelets are defined as subsequences containing information about the RUL. One can estimate the RUL based on the distance to them from the run-to-failure time series $x_{1:t}$ (Malinowski et al., 2015). More formally, the RUL shapelet is expressed as a tuple (s,δ,μ), where ***s*** is a subsequence, *δ* is a threshold for the distance between ***s*** and $x_{1:t}$, and *μ* is the estimated RUL. That is, we estimate RUL of $x_{1:t}$ as *μ* when ***s*** matches $x_{1:t}$ and $d\left(s,x_{1:t}\right)\leq \delta$.

Each element of the RUL shapelet is obtained as follows. ***s*** is one of the cluster centers obtained by applying the k-means clustering algorithm to every subsequence whose length is l=2,3,⋯,L, where *L* is the maximum length of RUL shapelets. Because ***s*** is a cluster center, one can say that it is close to other subsequences. The threshold *δ* is calculated as the minimum distance between ***s*** and the $i^{th}$ sample $x^{\left(i\right)}$ as presented in equation (4):

(4) $$\delta =\min_{t^{\prime }}d\left(s,x_{t^{\prime }:t^{\prime }+l}^{\left(i\right)}\right).$$

Let us define the RUL $\gamma^{\left(i\right)}$ after matching ***s*** to $x^{\left(i\right)}$ as shown in the equation (5):

(5) $$\gamma^{\left(i\right)}=T-{\mathrm{argmin}}_{t}\;d\left(s,x_{t:t+l}^{\left(i\right)}\right).$$

Let us also define ${\overset{˜}{\gamma }}^{\left(r\right)}$ be the *r*th smallest among $\gamma^{\left(i\right)}$ for all *i*. Then, *μ* is the mean of $\left\{{\overset{˜}{\gamma }}^{\left(r\right)}|r=1,\;2,\;\cdots ,\;\overset{˜}{n}\right\}$, where $\overset{˜}{n}$ is calculated as presented in equation (6):

(6) $$\overset{˜}{n}={\mathrm{argmin}}_{2\leq r\leq n}\;Var\left[{\overset{˜}{\gamma }}^{\left(1\right)},{\overset{˜}{\gamma }}^{\left(2\right)},\cdots ,{\overset{˜}{\gamma }}^{\left(r\right)}\right],$$

where $Var\left[{\overset{˜}{\gamma }}^{\left(1\right)},\;{\overset{˜}{\gamma }}^{\left(2\right)},\cdots ,{\overset{˜}{\gamma }}^{\left(r\right)}\right]$ is the variance of ${\overset{˜}{\gamma }}^{\left(1\right)},\;{\overset{˜}{\gamma }}^{\left(2\right)},\cdots$, and ${\overset{˜}{\gamma }}^{\left(r\right)}$.

### 2.3 Genetic algorithm

GA is one of the most widely used metaheuristic algorithms to solve various time series data analysis problems. It was proposed early 1970s, but it is still powerful method and has been employed to solve recent research problems such as feature selection (Ahn and Hur, 2020), hyperparameter tuning (Ahn and Hur, 2020), shapelet selection (Xue et al., 2020), and so forth.

**Figure 1:**
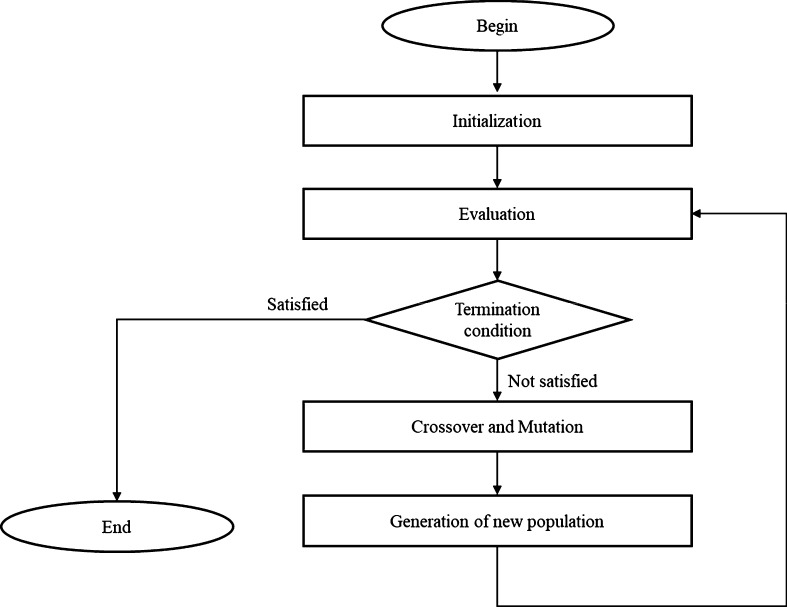
Optimization process using GA.

The optimization process using GA consists of four steps: (1) initialization, (2) evaluation, (3) crossover and mutation, and (4) generation of the new population. This process is presented in Fig. 1 (Vandewiele et al., 2021).

In the first step, a set of solutions called the population is initialized. In the second step, the solutions in the current population are evaluated using a fitness function, and some solutions with the highest fitness score are selected. In the third step, children of the selected solutions are generated using crossover and mutation operators. Crossover operators generate a child of two randomly selected solutions (called parents) and mutation operators add a variation to the child to avoid the situation where most solutions in the population are similar to each other. In the fourth step, the generated children and selected solutions compose a new population. If the termination condition is satisfied, then the currently best solution is returned; otherwise, steps (2) to (4) are repeated.

**Figure 2:**
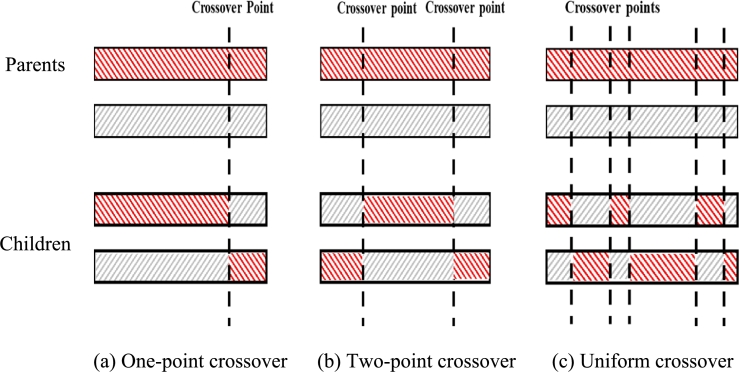
Popular crossover operators.

The solution representation method, crossover, and mutation operators in GA should be determined according to the specific purpose. For example, each solution can be represented as a binary vector for a feature selection problem (Ahn and Hur, 2020). As another example, each solution can be represented as a set of shapelets for a shapelet selection problem (Vandewiele et al., 2021). The most well-known crossover operators are one-point (see Fig. 2(a)), two-point (see Fig. 2(b)), and uniform crossover operators (see Fig. 2(c)). As seen in this figure, the crossover operators pick crossover points at random, and components of the parent solutions (i.e., genes) are swapped to generate children.

Examples of mutation operators are the flip bit operator, Gaussian operator, and so forth. The flip bit operator selects some genes at random and converts the genes into 1 if they are 0, and into 0 otherwise. Gaussian operators select some genes at random and add Gaussian noise to them.

## 3 Problem statement

Suppose we have run-to-failure data including *n* samples with different lengths $\left\{x^{\left(i\right)}|\;i=1,\;2,\;\cdots ,n\right\}$, where $x^{\left(i\right)}=\left(x_{1}^{\left(i\right)},x_{2}^{\left(i\right)},\cdots ,x_{T_{i}}^{\left(i\right)}\right)$ is the sample *i*. Here, $x_{t}^{\left(i\right)}\;\left(t=1,\;2,\;\cdots ,T_{i}\right)$ is the value measured immediately after time *t* from when the equipment described by the data started to be used. Additionally, $T_{i}$ is the lifetime of the equipment. Based on $T_{i}$, we can label RUL for all *i* and *t* as presented in equation (7):

(7) $$y_{t}^{\left(i\right)}=\frac{T_{i}-t}{T_{i}},$$

where $y_{t}^{\left(i\right)}$ is the label for $x_{t}^{\left(i\right)}$. We use the relative RUL instead of absolute RUL (i.e., $T_{i}-t$) for effective machine learning modeling. Using the label, we convert the run-to-failure data into a training dataset *D* with the following equation (8):

(8) $$D=\left\{\left(x_{1:t}^{\left(i\right)},y_{t}^{\left(i\right)}\right)|i=1,2,\cdots ,n;t=1,2,\cdots ,T_{i}\right\},$$

where $x_{1:t}^{\left(i\right)}$ denotes $\left(x_{1}^{\left(i\right)},x_{2}^{\left(i\right)},\cdots ,x_{t}^{\left(i\right)}\right)$. We use $x_{1:t}^{\left(i\right)}$ instead of $x_{t}^{\left(i\right)}$ to extract cumulative information until *t* to predict RUL at *t*.

The problem considered in this paper is to select a set of RUL shapelets, $S=\left\{s_{1},s_{2},\cdots ,s_{m}\right\}$, from *D* with three objectives: (1) to minimize the error of RUL prediction, (2) to minimize the number of RUL shapelets, and (3) to minimize redundancy among the shapelets. We define RUL shapelets as subsequences whose distances to $x_{1:t}^{\left(i\right)}$ are used as a feature vector for ML- or DL-based RUL prediction models. In other words, we predict $y_{t}^{\left(i\right)}$ with a trained regression model *f* as presented in equation (9):

(9) $${\overset{ˆ}{y}}_{t}^{\left(i\right)}=f\left(d\left(s_{1},\;x_{1:t}^{\left(i\right)}\right),d\left(s_{2},\;x_{1:t}^{\left(i\right)}\right),\cdots ,d\left(s_{m},\;x_{1:t}^{\left(i\right)}\right)\right).$$

The three objectives can be mathematically expressed as shown in equation (10), (11), and (12), respectively:

(10) $$\mathrm{minimize}\frac{1}{\sum_{i=1}^{n}T_{i}}\sum_{i=1}^{n}\sum_{t=1}^{T_{i}}\left|y_{t}^{\left(i\right)}-{\overset{ˆ}{y}}_{t}^{\left(i\right)}\right|,$$

(11) minimizem,

(12) $$\mathrm{minimize}\sum_{j=1}^{m-1}\sum_{k=j+1}^{m}\rho \left(d\left(s_{j},\;x_{1:t}^{\left(i\right)}\right),d\left(s_{k},\;x_{1:t}^{\left(i\right)}\right)\right),$$

where ρ(⋅) is the Pearson correlation coefficient, which is adopted because it is frequently-used to measure the redundancy among the features of a supervised model (Nasir et al., 2020) and the RUL shapelets are also the features. In addition to these three objectives, the efficiency of exploring *S* should also be considered. That is, we cannot solve the problem by comparing all possible candidates *S* due to the large search space (i.e., the number of all possible candidate *S* is $2^{\sum_{i=1}^{n}\frac{T_{i}\times \left(T_{i}-1\right)}{2}}$ in the worst case scenario).

## 4 Proposed algorithm

**Figure 3:**
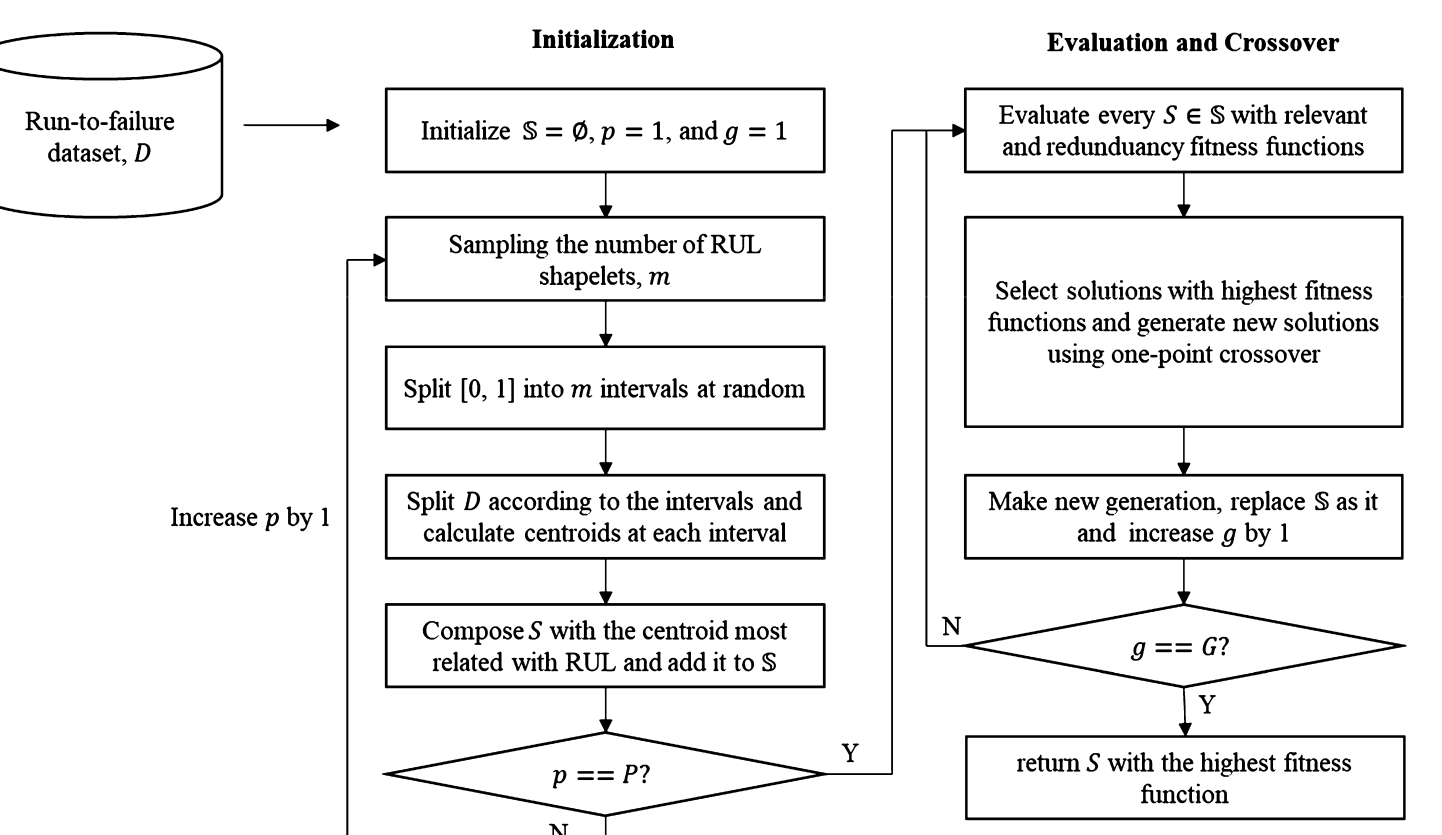
Overall flow chart of the proposed algorithm.

This section proposes the algorithm to compose a set of RUL shapelets using GA. The overall flow chart to select RUL shapelets is illustrated in Fig. 3 and Algorithm 1.

**Algorithm 1:**
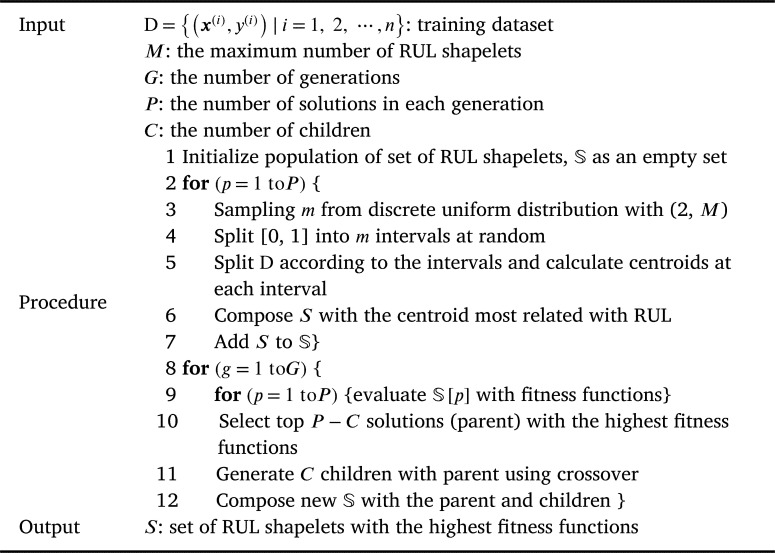
Overall pseudocode of the proposed Algorithm

Solution of the proposed algorithm is a set of RUL shapelets and the proposed algorithm selects the solution by generating and evaluating many solution candidates. The specific process is as follows. As the first step, it generates *P* initial solutions as follows.

(1) *m* is sampled from discrete uniform distribution whose lower bound is 2 and upper bound is *M*

(2) range [0, 1] is split into *m* intervals such as [0, 0.3), [0.3, 0.6), [0.6, 1.0] when m=3

(3) data is divided according to the intervals. For example, the data whose RUL is between 0.3 and 0.6 is included in [0.3, 0.6).

(4) For each interval, centroid $C_{k}$ with length k(k=2,3,⋯,K), is calculated and the centroid which has the highest correlation with RUL is selected as a RUL shapelet. By doing this, the lengths of RUL shapelets in the same solution become different from each other.

In the second step, *P* solutions are evaluated using two fitness functions (i.e., relevant function and a redundancy fitness function) and P−C solutions with the highest fitness are selected. We call them parents. In the third step, two parents are selected at random and a child is generated using a crossover operator. It repeats *C* times and after that new generation with P−C parents and *C* children is composed. Finally, the algorithm repeats the second and third steps, and returns the best solution ever found during the iterations.

**Figure 4:**
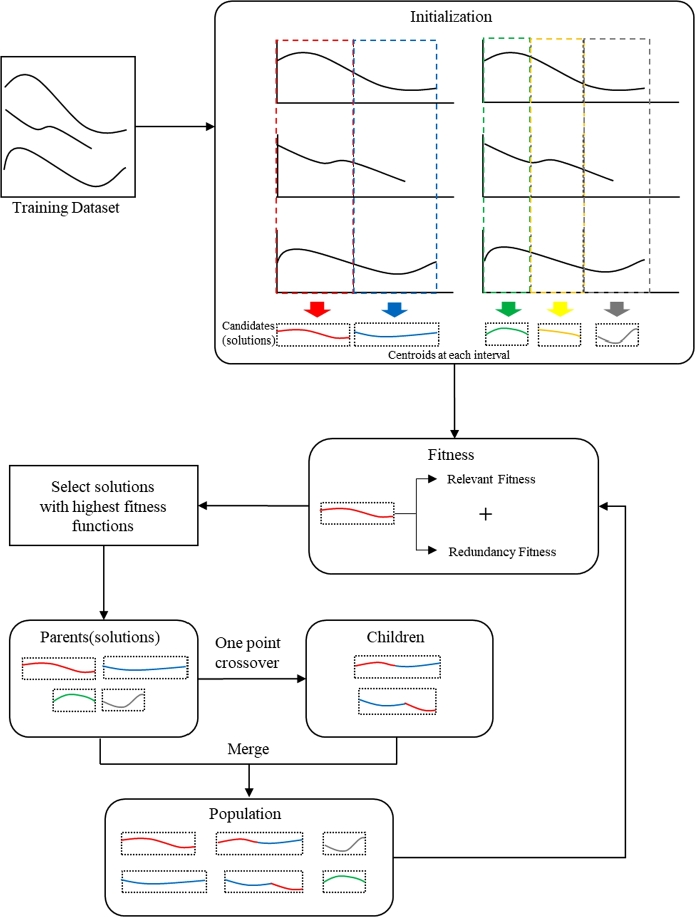
Visualization of the proposed algorithm.

Fig. 4 visualizes the proposed algorithm for the reader's understanding.

### 4.1 Properties of good RUL shapelets

Before describing the proposed method in detail, we discuss some properties of good RUL shapelets. First, even though the distances to RUL shapelets and RULs should be correlated, selecting RUL shapelets based on this correlation may lead to focusing on the later part of the time series due to the distance property presented in equation (13):

(13) $$d\left(s,x_{1:t}^{\left(i\right)}\right)\geq d\left(s,x_{1:T}^{\left(i\right)}\right)\;\text{if}t\leq T.$$

In other words, for any two time points $t_{f}$ and $t_{r}$ with $t_{r}>t_{f}$, it would be rare for the subsequence $s_{f}$ that occurs at $t_{f}$ to have a higher correlation with RUL than a subsequence $s_{r}$, which usually occurs at $t_{r}$. This is the case because $d\left(s_{f},x_{1:t}^{\left(i\right)}\right)$ is not dramatically decreased after $t_{f}$, while $d\left(s_{r},x_{1:t}^{\left(i\right)}\right)$ is. Since using sets of RUL shapelets that usually occur only at the rear is not appropriate for the RUL prediction, we should consider both the location of RUL shapelets as well as the correlation with the RUL.

**Figure 5:**
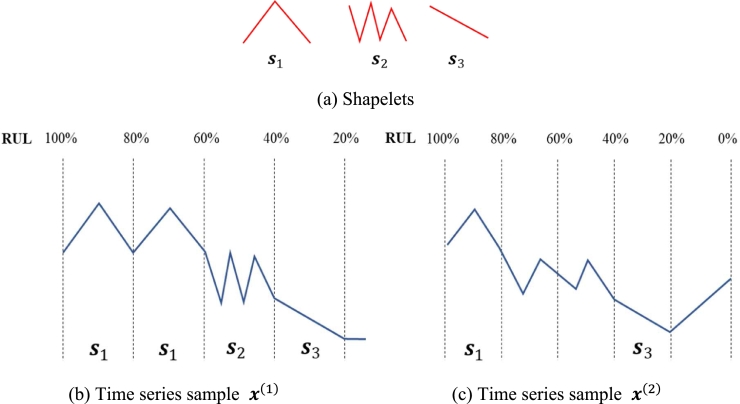
Example of good and bad RUL shapelets.

Second, good RUL shapelets should occur at similar locations in every time series sample. Fig. 5 illustrates an example of good and bad RUL shapelets with two time series samples $x^{\left(1\right)}$ and $x^{\left(2\right)}$ and three RUL shapelets $s_{1}$, $s_{2}$, and $s_{3}$. As seen in this figure, $s_{1}$ occurs at the interval [100%, 80%] of both samples, but also appears at [80%, 60%] in $x^{\left(1\right)}$. $s_{2}$ occurs in the first sample only and $s_{3}$ occurs at the interval [40%, 20%] in both samples but does not occur at any other intervals. Therefore, we can say that $s_{1}$ and $s_{2}$ are bad RUL shapelets, but $s_{3}$ is a good shapelet.

Third, two or more good RUL shapelets in the same interval causes redundancy, which may decrease the RUL prediction accuracy of a supervised model. On the contrary, good RUL shapelets from different intervals will interact with each other to increase accuracy.

**Figure 6:**
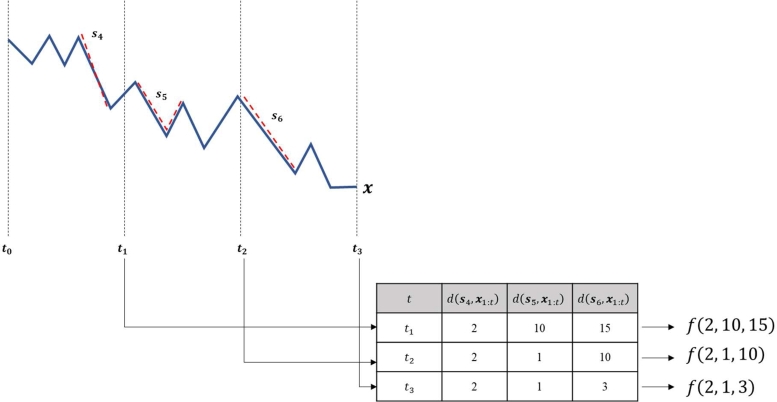
RUL prediction process with a good set of RUL shapelets.

We explain the RUL prediction process with good RUL shapelets $s_{4},\;s_{5}$, and $s_{6}$ at time $t\in \left\{t_{1},\;t_{2},\;t_{3}\right\}$, as presented in Fig. 6.

In this figure, a red-dashed line means an RUL shapelet is matched to the corresponding subsequence. For example, $s_{1}$ is matched to the subsequence in the interval $\left[t_{0},\;t_{1}\right)$ and its distance is 2. The distances to RUL shapelets at each time are used as a feature vector of regression model *f*. For example, the distances are 2, 10, and 15 at $t_{1}$. Accordingly, (2,10,15) is used as the feature vector. Note that the distance to each shapelet is not changed after the matching due to the property described in equation (13). For example, $d\left(s_{1},x_{1:t}\right)$ is not changed after $s_{1}$ matches the subsequence in the interval $\left[t_{0},\;t_{1}\right)$. Note also that one cannot exactly know whether a shapelet is matched or not until the entire time series sample is observed.

### 4.2 Initialization

The properties of a good RUL shapelet can be summarized as follows. First, its distance to the run-to-failure time series samples is highly correlated with the RUL. Second, it occurs at similar intervals in most time series samples. Third, it does not occur together with other RUL shapelets in the same interval. Based on these properties, we develop an initialization method for the proposed GA algorithm.

An offspring (i.e., a solution) of the proposed algorithm is a set $S=\left\{s_{1},\;s_{2},\;\cdots ,\;s_{m}\right\}$ of RUL shapelets. The initialization process of *S* using a training dataset $D=\left\{\left(x^{\left(i\right)},y^{\left(i\right)}\right)|i=1,\;2,\;\cdots ,n\right\}$ is described as follows.

First, the number of RUL shapelets, *m*, is sampled from the discrete uniform distribution DU(2,M), where M is a user parameter indicating the maximum number of RUL shapelets. Second, [0, 1] is split into *m* intervals at random as shown in the equation (14):

(14) $$V=\left\{\left[0,\frac{1}{m}+e_{1}\right),\left[\frac{1}{m}+e_{1},\frac{2}{m}+e_{2}\right)\cdots ,\left[\frac{m-1}{m}+e_{m-1},1\right]\right\},$$

where V is a set of intervals and $e_{j}$ is a random variable that follows a continuous uniform distribution CU(−0.1,0.1). Third, D is split into each interval as presented in the equation (15):

(15) $$D_{j}=\left\{\left(x_{s_{i}:e_{i}}^{\left(i\right)},y_{s_{i}:e_{i}}^{\left(i\right)}\right)|s_{i}=\left\lfloor T_{i}\times V_{j}\left[0\right]\right\rfloor ,e_{i}=\left\lceil T_{i}\times V_{j}\left[1\right]\right\rceil ,\forall i\right\},$$

where the data are split into intervals and $D_{j}$ is the *j*th interval, $V_{j}$ is the *j*th interval in V (i.e., $\left[\frac{j-1}{m}+e_{j-1},\frac{j}{m}+e_{j}\right)$, and $V_{j}\left[0\right]$ and $V_{j}\left[1\right]$ are the lower bound and upper bound of $V_{j}$, respectively.

Fourth, the centroid $c_{j,k}$ of a subsequence with length 2≤k≤K for every $D_{j}$ is calculated as shown in the equation (16):

(16) $$c_{j,k}=\frac{1}{n}\times \sum_{i=1}^{n}\frac{\sum_{t=s_{i}}^{e_{i}-k}x_{t:t+k}^{\left(i\right)}}{e_{i}-s_{i}-k},$$

where *K* is a user parameter indicating the maximum length of RUL shapelets. Finally, the centroid whose distance to the time series is the most correlated with the RUL is selected and used as $s_{j}$ for all *j* as presented in equation (17):

(17) $$s_{j}={\mathrm{argmax}}_{k}\;\rho \left({\left(d\left(x_{1:t}^{\left(i\right)},c_{j,k}\right)\right)}_{i,t},{\left(y_{t}^{\left(i\right)}\right)}_{i,t}\right).$$

Here, ${\left(d\left(x_{1:t}^{\left(i\right)},c_{j,k}\right)\right)}_{i,t}$ is a vector $\left(d\left(x_{1:k}^{\left(1\right)},c_{k}\right),d\left(x_{1:k+1}^{\left(1\right)},c_{k}\right),\cdots ,d\left(x_{T_{n}-k:T_{n}}^{\left(n\right)},c_{k}\right)\right)$.

The whole process is summarized in Algorithm 2, which is repeated *P* times, where *P* is the population size which is the number of solutions in each generation.

**Algorithm 2:**
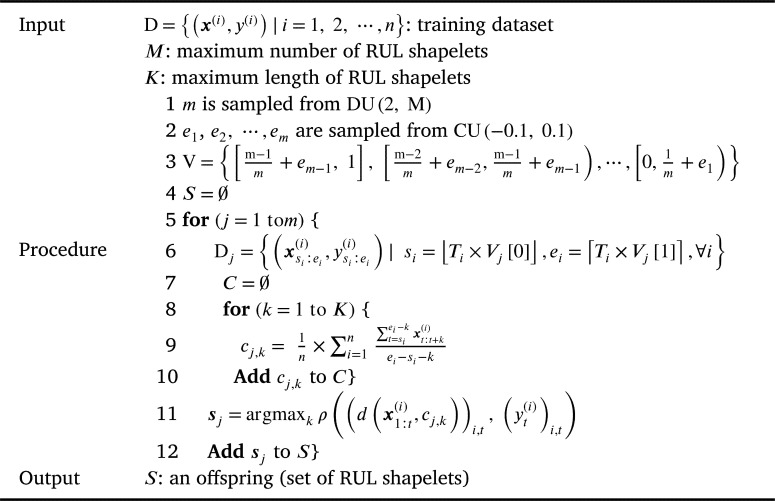
Population Initialization

### 4.3 Evaluation and crossover

Based on the properties of good RUL shapelets, we propose two fitness functions: a relevant function and a redundancy fitness function. The former evaluates an offspring $S=\left\{s_{1},\;s_{2},\;\cdots ,\;s_{m}\right\}$ in terms of the correlation between RUL and the minimum cosine distance of $s_{j}\;\left(j=1,\;2,\;\cdots ,m\right)$ to $x_{1:t}^{\left(i\right)}$. To be more specific, let $\theta_{j,t}^{\left(i\right)}$ be the minimum cosine distance between $x_{1:t}^{\left(i\right)}$ and $s_{j}$, which is obtained as shown in the equation (18):

(18) $$\theta_{j,t}^{\left(i\right)}=\min_{\tau =1,2,\cdots ,t-l}COS\left(s,x_{\tau :\tau +l}^{\left(i\right)}\right),$$

where $COS\left(s,x_{t:t+l}^{\left(i\right)}\right)$ is the cosine distance between ***s*** and $x_{t:t+l}^{\left(i\right)}$. Here, we use the cosine distance instead of other distance measures such as Euclidean distance to calculate the shape similarity, because the degradation patterns of RUL shapelets are, in general, more related with shapes than values. The relevant fitness function value $f_{1}(S)$ of *S* is calculated as the absolute mean of the Pearson correlation coefficient between $\theta_{j}={\left(\theta_{j,t}^{\left(i\right)}\right)}_{i,\;t}$ and $y={\left(y_{t}^{\left(i\right)}\right)}_{i,\;t}$ as presented in the equation (19):

(19) $$f_{1}(S)=\frac{1}{m}\times \sum_{j=1}^{m}\left|\rho \left(\theta_{j},y\right)\right|,$$

where *ρ* is the Pearson correlation coefficient.

The redundancy fitness function value $f_{2}(S)$ of *S* evaluates an offspring in terms of the correlation between the feature value $c_{j,t}^{\left(i\right)}$. That is, *S* is evaluated as shown in the equation (20):

(20) $$f_{2}\left(S\right)=\frac{2}{m\left(m-1\right)}\times \sum_{j=1}^{m-1}\sum_{j^{\prime }=j+1}^{m}\left|\rho \left(d_{j},d_{j^{\prime }}\right)\right|,$$

where $d_{j}$ is $d{\left(s_{j},\;x_{1:t}^{\left(i\right)}\right)}_{i,\;t}$.

Every offspring *S* is evaluated as $f_{1}(S)\times f_{2}\left(S\right)$. Some offspring in the population with the highest evaluation score are selected, and new offspring are generated using one-point crossover, as illustrated in Fig. 2 (a).

## 5 Experiment

In this section, we conduct three experiments to validate the effectiveness of our method and show how to use it properly.

### 5.1 Experimental design

In the first experiment, we compare the proposed method with other similarity-based methods by applying them to several benchmark datasets. The methods used in the experiment are Lyu et al. (2020)'s, Liu et al. (2019)'s, Bingjie et al. (2021)'s and Malinowski et al. (2015)'s, which are similarity-based methods. Please refer to the fifth paragraph in the Introduction section for the brief explanation on these methods. Their hyperparameters are summarized in Table 1.

**Table 1:** The methods used in this experiment.

Study	Hyperparameters	Values
Ours	Maximum length of RUL shapelets (*K*)	5, 10, 15, 20
	Maximum number of RUL shapelets (*M*)	5, 10, 15, 20
	Number of children (*C*)	3, 5, 7
	Number of generations	10
	Number of offspring in a population	10
	Model	DT, RF, LR, Lasso, SVR, MLP, KNN
Lyu et al. (2020)	Window size (w)	5, 10, 15, 20
	Window size variation (Δw)	0, 1, 2
	Number of neighbors	5, 10, 15, 20
	Scaling factor (*α*)	0, 300, 600, 900, 1200
	Scaling factor (*β*)	0, 30, 60, 90, 120
	Scaling function s(x)	$\frac{2}{1+e^{-x}}-1$, $\frac{x}{1+\left|x\right|}$, $\frac{x}{\sqrt{1+x^{2}}}$, tanh⁡(x)
Liu et al. (2019)	Window size	5, 10, 15, 20
	Weight of Euclidean similarity (*α*)	1
	Weight of cosine similarity (*β*)	1
Bingjie et al. (2021)	Number of windows	5, 6, 7, 8, 9
	Number of clusters	5, 10, 15, 20
Malinowski et al. (2015)	Maximum length of RUL shapelets	5, 10, 15, 20
	Number of centroids	50, 100, 150, 200, 250, 300
	Distance threshold	0.15, 0.25, 0.35, 0.45, 0.55

* DT: Decision Tree; RF: Random Forest; LR: Linear Regression; Lasso: Least Absolute Shrinkage and Selection Operator (α=0.2); SVR: Support Vector Regression; MLP: Multiple Layer Perceptron; KNN: K-Nearest Neighbors.

We compare every method with every combination of hyperparameters presented in Table 1 in terms of the prediction score (PS) proposed by Saxena and Goebel (2008) for each run-to-failure dataset. The prediction score is defined as presented in the equation (21).

(21) $$PS\left(y_{t},{\overset{ˆ}{y}}_{t}\right)=\left\{ \begin{array}{c} \exp\left(\frac{{\overset{ˆ}{y}}_{t}-y_{t}}{10}\right)-1,\;\text{if }y_{t}\leq {\overset{ˆ}{y}}_{t}\\ \exp\left(\frac{y_{t}-{\overset{ˆ}{y}}_{t}}{13}\right)-1,\;\text{if }y_{t}>{\overset{ˆ}{y}}_{t} \end{array}\right.,$$

where $y_{t}$ is an actual RUL and ${\overset{ˆ}{y}}_{t}$ is a predicted RUL. The specific procedure using a dataset, which consists of a training dataset and a test dataset, is described as follows. First, we convert every sample in the dataset into the structure presented in equation (8). Second, we train a model with a specific hyperparameter combination *h* and calculate the prediction score for test sample $i^{\prime }\;\left(i^{\prime }=1,\;2,\;\cdots ,\;n^{\prime }\right)$, where $n^{\prime }$ is the number of test samples as determined in equation (22):

(22) $$PS_{h,i^{\prime }}=\frac{1}{\left\lfloor T_{i^{\prime }}\times 0.9\right\rfloor -\left\lfloor T_{i^{\prime }}\times 0.2\right\rfloor }\times \sum_{t=\left\lfloor T_{i^{\prime }}\times 0.2\right\rfloor }^{\left\lfloor T_{i^{\prime }}\times 0.9\right\rfloor }PS\left(y_{t}^{\left(i^{\prime }\right)},{\overset{ˆ}{y}}_{h,t}^{\left(i^{\prime }\right)}\right).$$

Here, ${\overset{ˆ}{y}}_{h,\;t}^{\left(i^{\prime }\right)}$ is the value of $y_{t}^{\left(i^{\prime }\right)}$ predicted using the model with *h*. We do not use $x_{1:t}^{\left(i\right)}$ when $t<\left\lfloor T_{i}\times 0.2\right\rfloor$ or $t>\left\lfloor T_{i}\times 0.9\right\rfloor$ because it may be useless to estimate RUL if *t* is too small or too large. The PS for each method is calculated as shown in the equation (23):

(23) $$PS=\frac{1}{n^{\prime }\times \left|H\right|}\sum_{h\in H}\sum_{i^{\prime }=1}^{n^{\prime }}PS_{h,i^{\prime }},$$

where *H* is the set of all possible hyperparameter combinations and |H| is its size. Note that we calculate *PS* 10 times for methods with randomness and use their average for the objective comparison.

In the second experiment, we validate the initialization algorithm of the method by comparing it with random initialization algorithm employed in most GAs. The random initialization algorithm generates m≤M initial RUL shapelets by selecting a sample from the training dataset and randomly choosing a subsequence whose length is smaller than *K*.

In the third experiment, we conduct sensitivity analysis on the parameters to show the relationship between hyperparameters and the RUL prediction accuracy. We also suggest how to determine the parameters. We conduct repeated measures analysis of variance (RMANOVA) with data when the independent variables are the hyperparameters *K*, *M*, *C*, and Model and the dependent variable is the mean MAE of the model under the hyperparameters for each dataset to find the most important hyperparameters. Then, we conduct the sensitivity analysis for the selected important hyperparameters, leaving other important parameters fixed.

### 5.2 Datasets

The datasets for the experiments are C-MAPSS (commercial modular aero-propulsion system simulation) datasets provided by Saxena and Goebel (2008). These are simulation datasets obtained under several operating conditions and fault modes, and each of them can be distinguished according to the number of conditions and modes. Chao et al. (2021) use C-MAPSS to generate more realistic simulation datasets, considering real flight condition. We call the dataset provided by Saxena and Goebel (2008) C-MAPSS (ver.1) and the dataset provided by Arias Chao et al. C-MAPSS (ver 2). Each dataset has 14 health parameters, and we make a new health index that is used as a time series to predict RUL, as presented in Zhu et al. (2021). The other datasets are life time of Li-ion batteries measured under various room temperature provided by Goebel et al. (2008). The datasets are separated according to its operating conditions, but we combined all because each dataset has few data and similarity based methods can handle the combined data. We call the dataset Battery.

Specific information on the datasets is summarized in the following Table 2.

**Table 2:** Datasets used in the experiments.

Dataset	Number of	Number of	Mean length of	Mean length of
	training samples	test samples	training samples	test samples
C-MAPSS (ver.1) #1	75	25	210.16	194.76
C-MAPSS (ver.1) #2	195	65	206.68	207.03
C-MAPSS (ver.1) #3	75	25	242.84	260.28
C-MAPSS (ver.1) #4	186	63	247.54	241.38
C-MAPSS (ver.2) #1	6	3	75.83	64.33
C-MAPSS (ver.2) #2	11	4	73.55	73.00
C-MAPSS (ver.2) #3	11	4	67.18	63.75
Battery	23	8	83.26	100.50

As seen in this Table 2, each dataset consists of training and test dataset and C-MAPSS data consists of several datasets according to operating conditions such as fault mode. We will call each dataset in a row of the table dataset if there is no confusion in meaning and thus we have eight datasets.

### 5.3 Results

**Figure 7:**
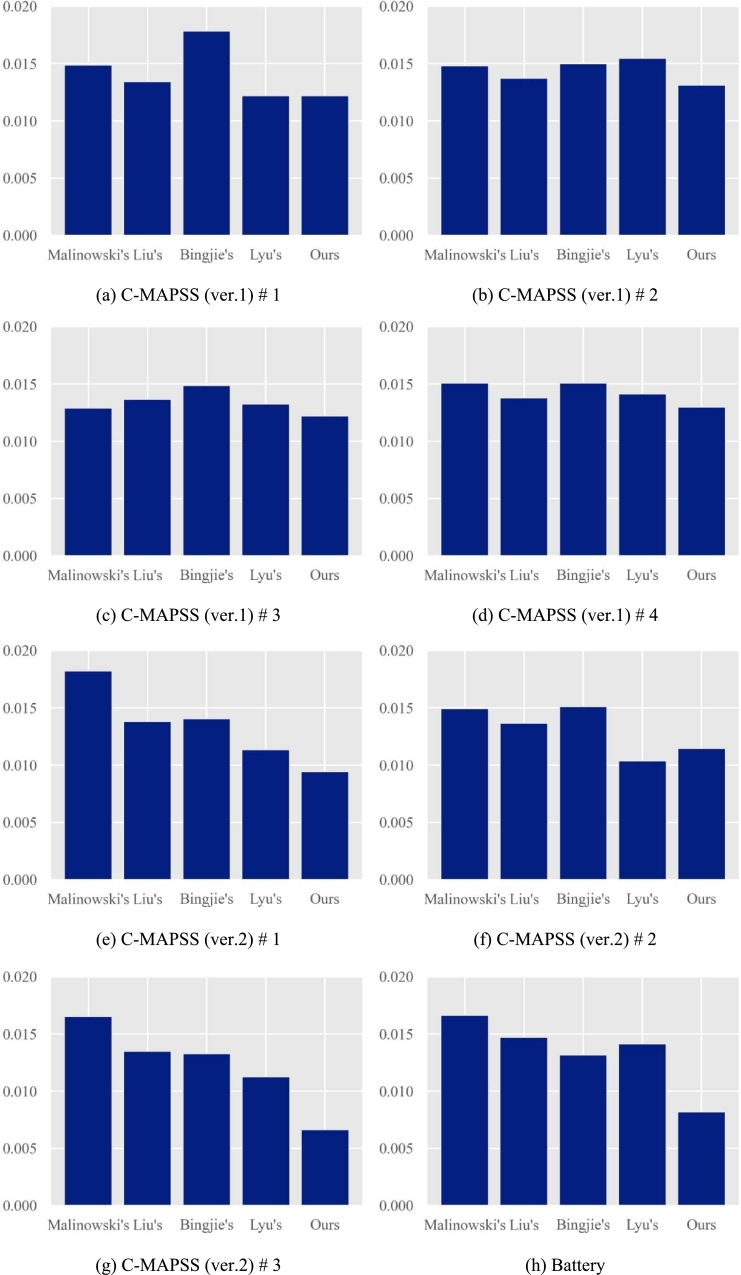
Comparison results.

Fig. 7 (a)-(h) compares the mean PSs of each method in each dataset.

As seen in this figure, for the most datasets, our method outperforms the other methods, especially the method of Malinowski et al. (2015) that first introduced RUL shapelets. As we mentioned earlier, Malinowski et al. (2015) ignored the rule that good RUL shapelets should be frequent only in the specific range when developing a method. Our proposed method took this characteristic of good shapelets, as presented in subsection 4.1, into consideration. As a result, our performance was improved. To be more concrete, our method outperforms the method proposed by Malinowski et al. (2015) for all the eight benchmark datasets. In addition, our method also outperforms previous similarity-based methods for most datasets. Average PS of our method is bigger than Lyu's method only for two datasets, as seen in (a) c-MAPSS (ver.1) #1 and (f) c-MAPSS (ver.2) #2. Average PS of our method is bigger than Lyu's only for two datasets (a) c-MAPSS (ver.1) #1 and (f) c-MAPSS (ver.2) #2. Our method gives the second-best performance, which seems to come from the lack of data integrity or the noise in the dataset during the model training.

**Figure 8:**
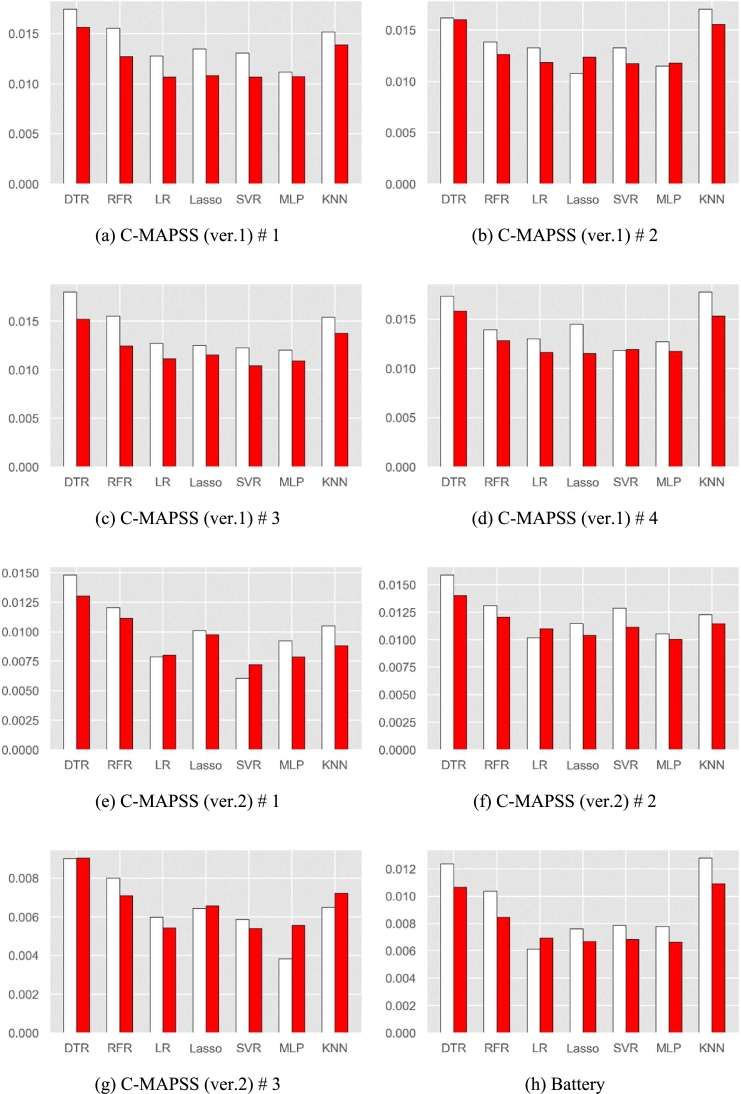
Comparison result between the proposed and random initialization methods.

Fig. 8 compares the mean PSs between the proposed initialization method and random initialization method for several regression models. In this figure, left white bar and right red bar are average PS using the random and proposed initialization methods, respectively.

From the figure, we can conclude that the proposed initialization method is better than the random initialization especially when the run-to-failure data is large. Specifically, the average PS's of our initialization method are smaller than those of random initialization for most datasets and regression models, except in 5 results among 56 results (eight datasets and seven regression models).

Table 3 shows the RMANOVA results where each cell denotes F-value (p-value). For example, F-value and p-value of parameter *M* for data C-MAPSS (ver.1) #1 are 1.08 and 0.3599, respectively.

**Table 3:** RMANOVA results.

Data	*M*	*C*	*K*	Model
C-MAPSS (ver.1) #1	1.08 (0.3599)	0.97 (0.3788)	420.29 (0.0000^⁎⁎^)	949.25 (0.0000^⁎⁎^)
C-MAPSS (ver.1) #2	3.48 (0.0165^⁎^)	0.79 (0.4568)	684.72 (0.0000^⁎⁎^)	2261.98 (0.0000^⁎⁎^)
C-MAPSS (ver.1) #3	0.54 (0.6546)	3.15 (0.0446^⁎^)	58.27 (0.0000^⁎⁎^)	99.88 (0.0000^⁎⁎^)
C-MAPSS (ver.1) #4	10.48 (0.0000^⁎⁎^)	2.42 (0.0913)	312.59 (0.0000^⁎⁎^)	128.05 (0.0000^⁎⁎^)
C-MAPSS (ver.2) #1	3.25 (0.0224^⁎^)	3.85 (0.0227^⁎^)	287.37 (0.0000^⁎⁎^)	57.72 (0.0000^⁎⁎^)
C-MAPSS (ver.2) #2	1.79 (0.1494)	1.52 (0.2204)	400.66 (0.0000^⁎⁎^)	437.44 (0.0000^⁎⁎^)
C-MAPSS (ver.2) #3	1.08 (0.3599)	0.97 (0.3788)	420.29 (0.0000^⁎⁎^)	949.25 (0.0000^⁎⁎^)
Battery	3.48 (0.0165^⁎^)	0.79 (0.4568)	684.72 (0.0000^⁎⁎^)	2261.98 (0.0000^⁎⁎^)

^⁎^: p-value < 0.05, ^⁎⁎^: p-value < 0.01

As seen in this table, the p-values for *K* and Model are smaller than 0.01 for every dataset. In particular, Model has the largest F-value except for C-MAPSS (ver.1) #4 and C-MAPSS (ver.2) #1. Thus, we conclude that the regression model is the most important parameter and the length of RUL shapelets is the second most important parameter.

**Figure 9:**
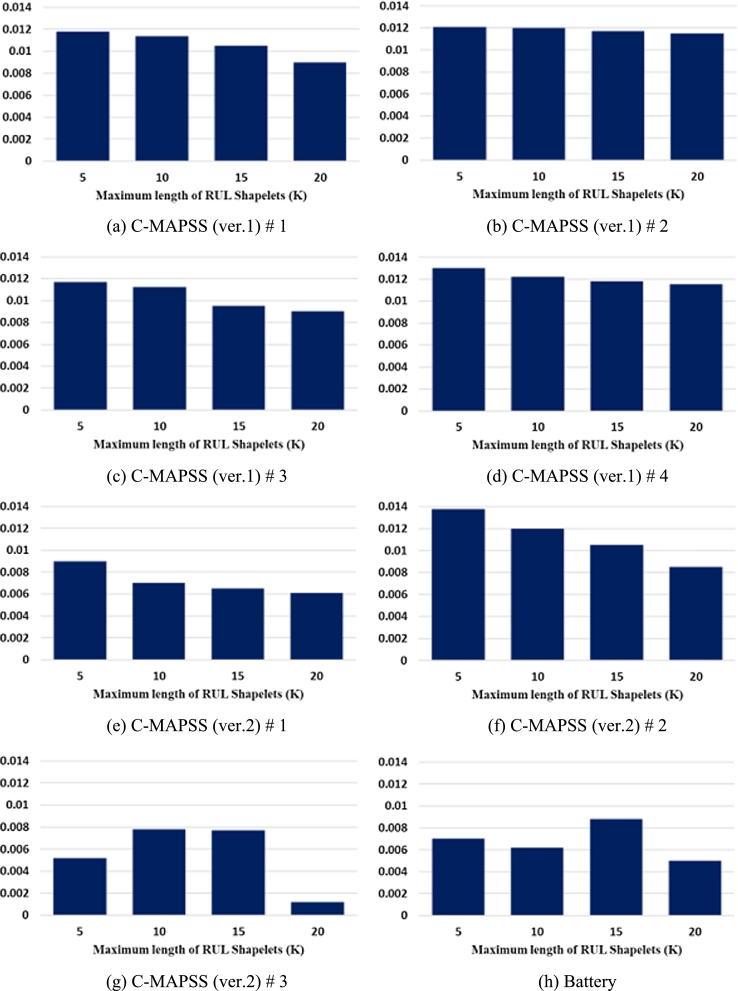
Sensitivity analysis results according to the length of RUL shapelets.

Fig. 9 shows the average PS obtained by sensitivity analysis for various K=5,10,15,20 when the model is SVR.

As seen in this figure, the larger the K is, the smaller the PS's are for all the datasets except for C-MAPSS (ver.2) #3 and Battery. Even for those two datasets, however, the PS's are the smallest when K = 20.

**Figure 10:**
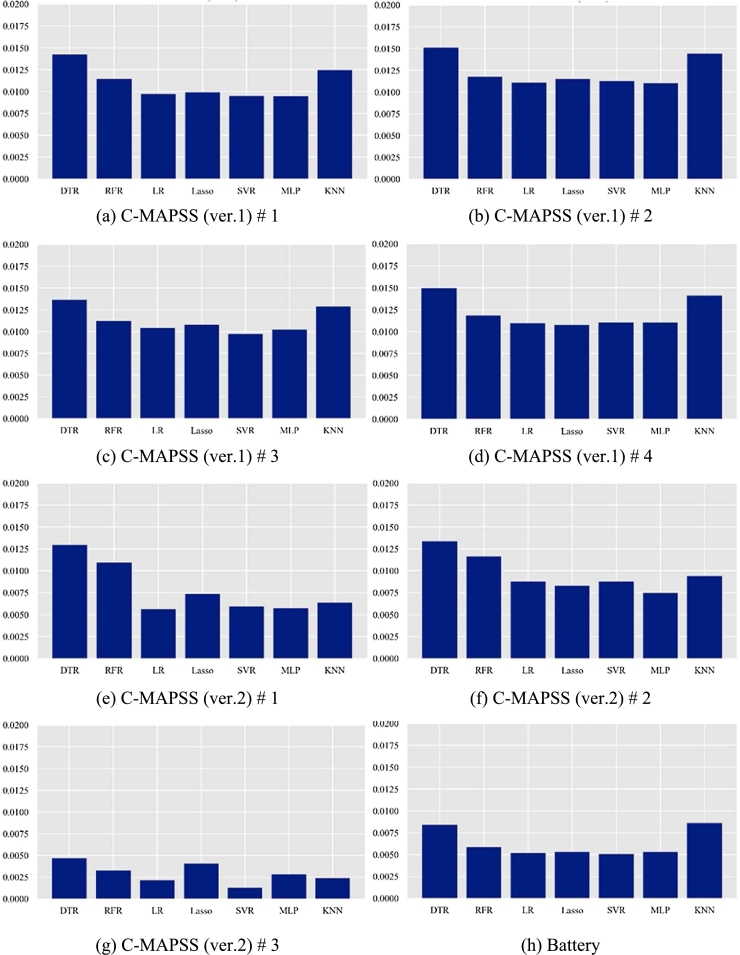
Sensitivity analysis results according to the regression model.

Fig. 10 shows the sensitivity analysis results according to the regression model when *K* is fixed as 20.

As seen in this figure, we can find that linear models, including Lasso, LR, SVR, and MLP, show better results than tree models like DT and RF.

The experimental results can be summarized as follows. First, our method outperforms previous similarity-based methods including the method of Malinowski et al. (2015) that first introduced RUL shapelets for most datasets. Second, our initialization method shows better results than those of random initialization method for most regression models and datasets. Third, the regression model and the length of RUL shapelets are the most and second most important parameters, respectively. Fourth, the larger the maximum length of RUL shapelets is, the smaller the RUL prediction errors are. Finally, the linear models such as Lasso, LR, SVR and MLP are more proper than tree models such as DT and RF.

## 6 Conclusion

In this paper, we formulized the RUL shapelet selection by using a mathematical optimization problem with three objectives: 1) to minimize the error of RUL prediction, 2) to minimize the number of RUL shapelets, and 3) to minimize redundancy among the shapelets. In addition, we characterized some of the properties that a good set of RUL shapelets should possess. First, the RUL of a time series sample is proportional to the minimum distance to each shapelet. Second, good RUL shapelets should occur at a similar location in every time series sample, while also not occurring at a different location. Finally, two or more shapelets should not occur in the same interval.

Based on these properties, we developed a GA-based RUL shapelet selection algorithm. This method selects frequent subsequences locally, not globally, and does not select two or more subsequences from the same interval. From our experiment, we validated that the proposed method outperforms previous methods. We also provided some guidelines for determining the hyperparameters and selecting the machine learning model. We also provided an initialization method that works well when the data is complicated or when the regression model is linear. And if we have a large number of RUL shapelets, we can get a smaller prediction error.

The limitations of the proposed methods are as follows. First, the method can only be used when a one-dimensional health index exists. In other words, the method works for univariate time series only. Second, the method is very expensive in terms of computational complexity. It requires iterative computation such as splitting the dataset based on intervals, finding centroids, crossover mutation for two sets of RUL shapelets and so forth. Finally, it is difficult to interpret the RUL prediction result when there are many RUL shapelets. Especially, this paper focuses only on the RUL prediction performance, and does not propose interpretation method using the selected RUL shapelets.

As for future research, we suggest the method should be expanded to the multivariate time series directly, without introducing the health index. We also suggest the approximation method to calculate the distance between time series and RUL shapelets, and the method to reduce the number of candidates for the fast search. Finally, the interpretation method is necessary to use the proposed method in practice.

## Declarations

### Author contribution statement

Gilseung Ahn: Conceived and designed the experiments; Analyzed and interpreted the data; Wrote the paper. Min-Ki Jin; Seok-Beom Hwang: Performed the experiments; Contributed reagents, materials, analysis tools or data. Sun Hur: Analyzed and interpreted the data; Wrote the paper.

### Funding statement

This research did not receive any specific grant from funding agencies in the public, commercial, or not-for-profit sectors.

### Data availability statement

Data will be made available on request.

### Declaration of interests statement

The authors declare no conflict of interest.

### Additional information

No additional information is available for this paper.
